# The association between gestational diabetes and ASD and ADHD: a systematic review and meta-analysis

**DOI:** 10.1038/s41598-021-84573-3

**Published:** 2021-03-04

**Authors:** Jennifer Rowland, Claire A. Wilson

**Affiliations:** 1 Faculty of Medicine, Dentistry and Health, University of Sheffield, Beech Hill Road, Sheffield, S10 2RX UK; 2 Section of Women’s Mental Health, King’s College London, PO31 King’s College London, London, SE5 8AF UK; 3 South London and Maudsley NHS Foundation Trust, Monks Orchard Road, Beckenham, London, BR3 3BX UK

**Keywords:** Risk factors, Endocrine system and metabolic diseases, Psychiatric disorders

## Abstract

There is growing evidence for a role of maternal diabetes in the pathogenesis of neurodevelopmental disorders. However, the specific association between gestational diabetes (GDM), as opposed to pre-gestational diabetes, has been poorly isolated. Thus the aim was to systematically review and meta-analyse literature pertaining to prevalence and risk for two neurodevelopmental disorders: autism spectrum disorder (ASD) and attention deficit hyperactivity disorder (ADHD), when exposed to GDM. PubMed, Cochrane Library, EMBASE, PsycINFO and CINAHL were systematically searched for eligible literature, with forward and backward citation tracking. Screening for eligibility, risk of bias assessment and data extraction were performed by two independent reviewers. 18 studies measuring ASD and 15 measuring ADHD met inclusion criteria. On meta-analysis there was an increased risk of ASD (OR 1.42; 95% CI 1.22, 1.65) but not ADHD (OR 1.01; 95% CI 0.79, 1.28). We discuss potential mechanisms for these differing risks. Greater understanding of risk factors, including GDM, for these neurodevelopmental disorders and potential mechanisms may help inform strategies aimed at prevention of exposure to these adversities during pregnancy.

## Introduction

Gestational diabetes (GDM) is glucose intolerance that begins during pregnancy and has an estimated prevalence of between 1.8% and 22.3% in Europe, with higher rates in Africa, North and South America and the Middle East^1^. It is associated with adverse outcomes for mother and baby, including obstetric complications such as emergency Caesarean delivery and longer-term risks of Type 2 Diabetes in the mother and metabolic syndrome in offspring^2^.

There is also some emerging evidence for a relationship between GDM and adverse neurobehavioural outcomes in children. Several systematic reviews suggest an association between maternal diabetes and lower IQ scores, language impairment and symptoms of attention deficit hyperactivity disorder (ADHD) and autism spectrum disorder (ASD). However, many of these reviews group together women experiencing pregestational (Type 1 and Type 2) and gestational diabetes so do not investigate the effect of GDM specifically^3–8^. While GDM and pregestational diabetes share similar pathology of insulin resistance, in GDM this insulin resistance arises only during pregnancy, which is itself a state of insulin resistance. Therefore, while there may be some women diagnosed with GDM who have undiagnosed pregestational diabetes, the pathology is slightly different between the two conditions. There are many potential mechanisms that may underpin such an association between GDM and adverse offspring neurobehavioural outcomes. There may be mediating factors of obstetric and neonatal adversities such as pre-eclampsia or infants born large for gestational age^9–11^. There may also be epigenetic changes^12^ or oxidative stress^13,14^ resulting from a hyperglycaemic in-utero environment.

Thus, the aim of this study was to conduct a systematic review and meta-analysis of the prevalence and risk for ADHD and ASD in children of women affected specifically by GDM. Both ADHD and ASD are commonly diagnosed neurodevelopmental disorders encompassing a spectrum of neurobehavioural symptoms that are often diagnosed from a young age. ADHD has a global prevalence of around 5%^15^, is characterised by symptoms of inattention and hyperactivity^16^ and often has broad and enduring adverse impacts on quality of life and functioning^17^. ASD describes a range of conditions characterised by some or all of: impaired communication, impaired social interaction and repetitive, restricted and stereotyped behaviour^16^ and may also result in profound struggles in both personal and professional life. A range of pathophysiological mechanisms have been implicated for these neurodevelopmental disorders, including hyperglycaemia during pregnancy^18^. Thus, a greater understanding of the aetiology of these disorders could help to identify early life risk factors for their development.

## Methods

The review followed ‘Meta-analysis of Observational Studies in Epidemiology’ (MOOSE)^19^ and ‘Preferred Reporting Items for Systematic Reviews and Meta-Analyses’ (PRISMA) guidelines^20^ and was registered with PROSPERO (CRD42019128376).

### Search strategy

An electronic literature search was performed in the databases PubMed, Cochrane Library, EMBASE, PsycINFO and CINAHL from inception to 04/04/2019, with forward and backward citation tracking of eligible papers. Search terms were adapted from previous systematic reviews in the area (see supplementary material). Two separate searches were conducted for ASD and ADHD.

### Study selection

Inclusion criteria were: published, peer-reviewed studies with children aged 18 and under, whose mothers had clinically diagnosed GDM during pregnancy and who were investigated for symptoms and/or diagnosis of ASD or ADHD. Report of symptoms of ASD and ADHD by questionnaires or other tests was accepted and clinical diagnosis was accepted through self-report, report from medical professionals or medical records. Either self-report of GDM, report from medical professionals, or medical records was accepted. Observational studies and baseline data from intervention studies were included, in any language.

Exclusion criteria were: case studies, editorials, reviews and conference abstracts. Non-human studies were also excluded. Studies which were known to include women with established pregestational diabetes were excluded, unless it was possible to extract data pertaining specifically to GDM. Studies in which there was some doubt surrounding this were included in the review but not included in the meta-analysis.

**Figure 1:**
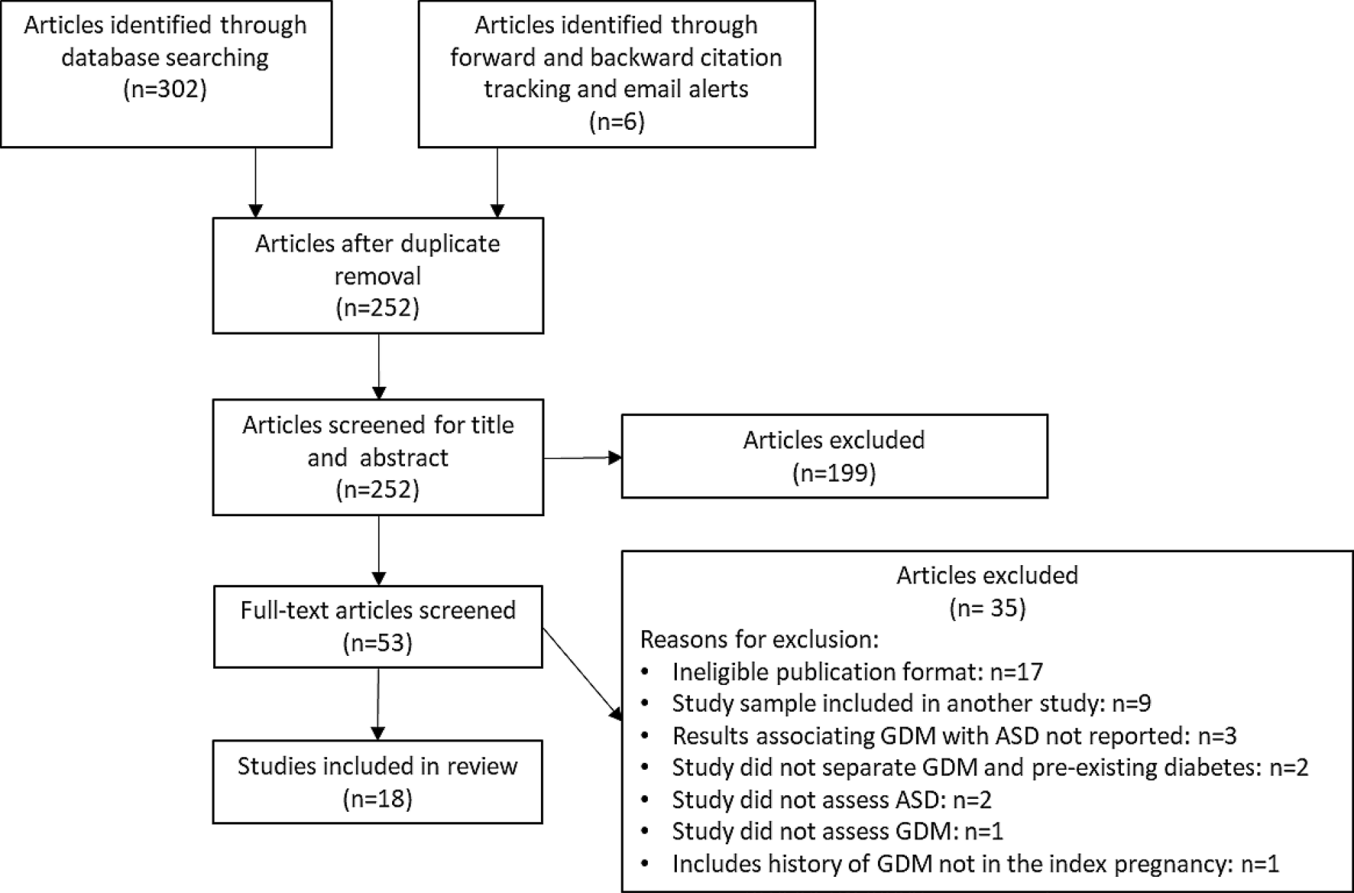
Flow diagram of ASD study selection.

**Figure 2:**
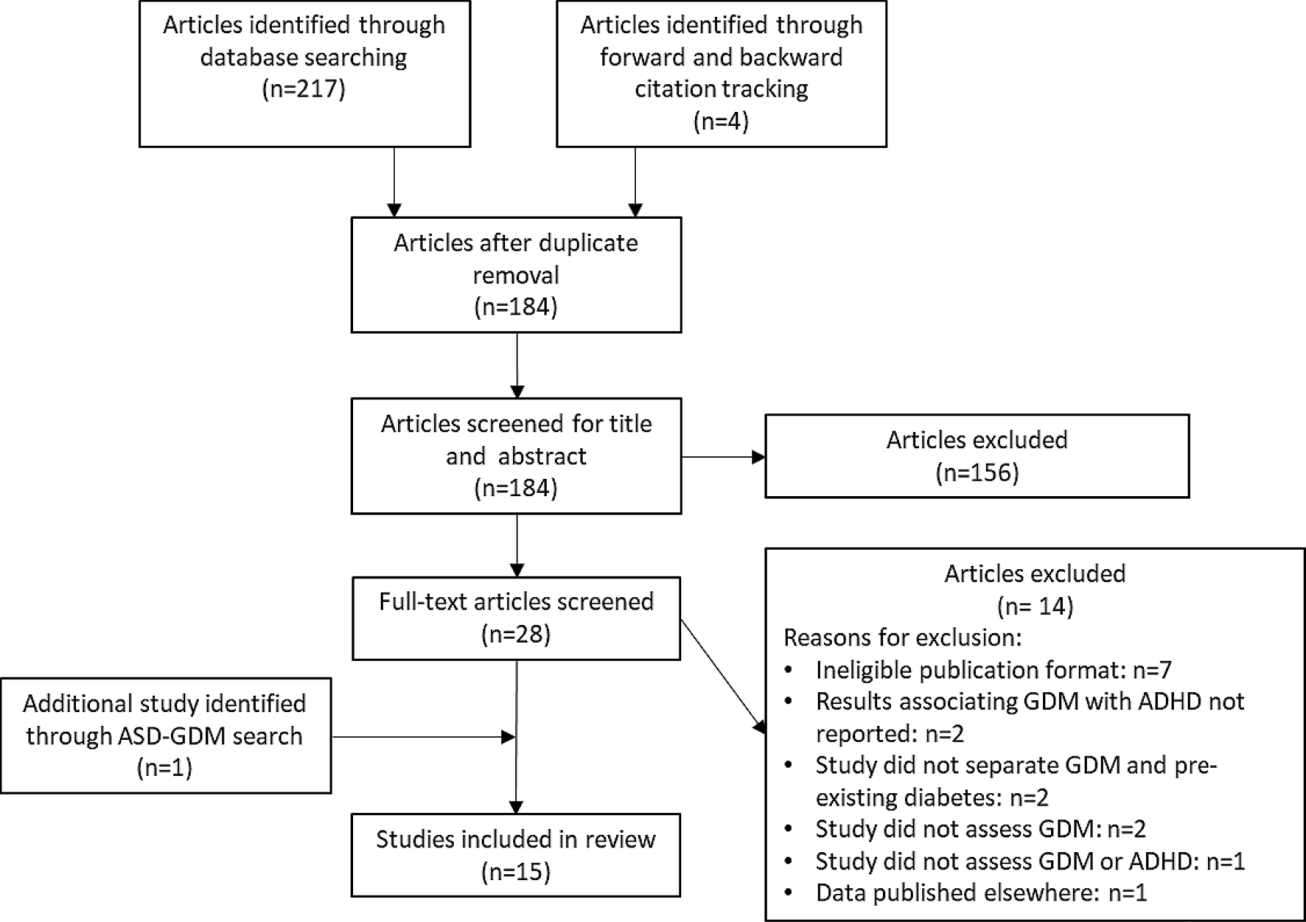
Flow diagram of ADHD study selection.

Two independent reviewers screened titles and abstracts then full texts for eligibility. Results of study selection are presented in Figs. 1 and 2.

### Data extraction and risk of bias assessment

Two independent reviewers extracted data, including study characteristics, prevalence and odds ratios (ORs) and any information on mechanisms for the associations. Study authors were e-mailed to request raw data if potentially relevant data may be available.

Risk of bias in all included studies was also assessed by two independent reviewers using a pre-piloted modified Newcastle Ottawa Scale (see supplementary material). Scores for selection bias and measurement bias were of particular interest as most of the studies were of observational design. Each question in the tool had a score of zero to two (low to high risk of bias). A score of two on any item within the selection and measurement bias domains meant that the study was categorised as having a high risk of bias.

### Data synthesis

Meta-analyses of ORs and prevalence were undertaken separately for ASD and ADHD if at least five studies were available^21^. In studies providing only prevalence data, ORs were calculated from this data (or raw data provided by authors). If there was any doubt as to whether or not pregestational diabetes had been excluded from the comparison group without GDM, these studies’ ORs were not included in meta-analysis.

Data were analysed using Stata 15. Metan and metaprop commands were used to produce pooled unadjusted ORs and prevalence and 95% confidence intervals (CIs) displayed as forest plots. Insufficient numbers of studies adjusted for similar characteristics to enable pooling of adjusted ORs. DerSimonian-Laird random effects meta-analysis^22^ was used as there was expected to be substantial heterogeneity between studies^23,24^. Heterogeneity was assessed using I^2^: proportion of total variation in study estimates that is due to heterogeneity^25^. It was decided a-priori that I^2^ > 75% would preclude meta-analysis as this represents considerable heterogeneity^26^. Both of the prevalence meta-analyses produced I^2^ > 75% so prevalence is presented as median with interquartile range (IQR) as a standard summary measure of non-parametric data. Sensitivity analyses on effect of risk of bias were conducted when sufficient studies were available. Publication bias was not assessed for the meta-analyses as there were insufficient numbers of studies (less than ten)^27^.

## Results

### Characteristics of ASD studies

18 studies measuring ASD were identified; three of these also measured ADHD. Table 1 provides a summary of their characteristics and findings. Nine of these studies were from North America. Four were from middle income countries; none were from low- income countries (according to World Bank classification at June 2019). All studies were observational. Most of the studies used medical records or parental report of ASD and GDM; diagnostic criteria for GDM were usually not reported. 11 of the studies were assessed as high risk of bias due to lack of information about how GDM or ASD was diagnosed, increasing the risk of measurement bias and/or lack of information about selection criteria preventing accurate assessment of risk of selection bias.

**Table 1:** Summary of studies measuring ASD.

Author and year	Study design	Country	Sample size and age of children at diagnosis	Ascertainment of GDM diagnosis	ASD measure	Findings [%, mean (SD), OR/HR/RR/β (95% CI)]	Risk of bias score (low–high: 0–2)	Risk of bias
Alshaban et al. 2019^28^	Cross-sectional	Qatar	176,960 children 844 with ASD and available clinical data Aged 5–12 years	Medical records or parental interviews Diagnostic criteria unknown	Parental report of diagnosis or formal assessment	8.9% of children with autism were exposed to GDM 1.14% of total study population had ASD	Selection bias: Representativeness: 0 Participation rates: 2 Measurement bias: GDM: 2 ASD: 0	High
Burstyn et al. 2010^50^	Prospective cohort (population-based)	Canada	7453 children of GDM mothers 206,122 children of non-diabetic mothers Median age 36 months (IQR 47–65)	Medical records Diagnostic criteria unknown	Diagnosis from medical records	0.72% of children of GDM mothers had autism 0.51% of children of non-diabetic mothers had autism RR: 1.24 (0.94–1.65)	Selection bias: Representativeness: 0 Participation rates: 0 Measurement bias: GDM: 1 ASD: 0	Low to moderate
Chien et al. 2018^51^	Retrospective cohort	Taiwan	323 children with ASD Mean age 10.7 years (SD 3.5) 257 unaffected siblings Mean age 11.7 years (SD 4.5) 1504 control children Mean age 8.9 years (SD 1.6)	Self-report, 15% cases validated with medical records Diagnostic criteria defined by Carpenter-Coustan: 2 positive values on 100 g oral glucose tolerance test (OGTT) 95 (0 h), 180 (1 h), 155 (2 h), 140 mg/dL (3 h)	Diagnosis from child psychiatrists	1.2% children with ASD had mothers with GDM 0.4% of unaffected siblings had mothers with GDM 0.7% of control children had mothers with GDM OR (ASD vs control): 1.78 (0.52–6.12) OR (ASD vs unaffected sibling): 3.42 (0.57–20.82) Beta coefficient for ASD symptom severity with GDM: 3.43 (SD = 1.32). (Adjusted for child sex and age)	Selection bias: Representativeness: 0 Participation rates: 2 Measurement bias: GDM: 2 ASD: 0	High
Connolly et al. 2016^41^	Retrospective cohort	USA	503 children with ASD 38,810 control children Age of children not reported	Medical records Diagnostic criteria unknown	Diagnosis from medical records	10.4% of children with ASD had mothers with GDM 6.6% of control children had mothers with GDM P = 0.0007 Unadjusted OR: 1.64 (1.22–2.22) Adjusted OR: 1.56 (1.14–2.11) (Adjusted for maternal age at birth, maternal race, year of birth and BMI) Sensitivity analysis restricting to births < 2011: OR: 1.74 (95% CI: 1.25–2.44) Adjusted OR: 1.44 (95% CI: 1.02–2.03) (adjusted for maternal age at birth, maternal race, year of birth and BMI)	Selection bias: Representativeness: 0 Participation rates: 0 Measurement bias: GDM: 1 ASD: 0	Low to moderate
Dodds et al. 2011^52^	Retrospective cohort	Canada	924 children with ASD 128,809 children without ASD Aged 1–17 years	Medical records Diagnostic criteria unknown	Diagnosis from medical records	0.9% of children of mothers with GDM had autism 0.7% of children of non-GDM mothers had autism RR: 1.29 (0.90–1.83) 0.7% of children of mothers with no diabetes had autism*	Selection bias: Representativeness: 0 Participation rates: 0 Measurement bias: GDM: 1 ADHD: 0	Low to moderate
George et al. 2014^53^	Case control	India	143 children with autism Mean age 42 months 200 control children Mean age 41.6 months	Self-report Diagnostic criteria unknown	Diagnosis using Child Autism Rating Scale (method of report unknown)	11.2% of children with autism had mothers with GDM 11.5% of controls had mothers with GDM	Selection bias: Representativeness: 1 Participation rates: 2 Measurement bias: GDM: 2 ASD: 0	High
Hadjkacem et al. 2016^54^	Cross-sectional	Tunisia	50 children with autism 51 control children Aged 3–7 years	Self-report Diagnostic criteria unknown	Diagnosis from child psychiatrist	8.0% of the autistic children had mothers with GDM 2.0% of the control children had mothers with GDM OR: 4.43 p = 0.2	Selection bias: Representativeness: 1 Participation rates: 2 Measurement bias: GDM: 2 ASD: 0	High
Kania et al. 2016^55^	Cross-sectional	Poland	1007 children of GDM women (no control group) Median age at diagnosis 4.5 years (range 2.5–7 years)	Medical records Diagnostic criteria from 1999- 2005: fasting glucose > 110 mg/dL or 140 mg/dL 2 h post OGTT From 2005–2011: fasting glucose > 100 mg/dL or > 140 mg/dL 2 h post OGTT	Diagnosis from parental report	0.08% of children were diagnosed with ASD	Selection bias: Representativeness: 1 Participation rates: 2 Measurement bias: GDM: 0 ASD: 1	High
Khanom et al. 2015^56^	Case control	Bangladesh	95 children with ASD 185 control children Aged 15–26 months	Self-report Diagnostic criteria unknown	Diagnosis Method of report unknown	40.4% of children with ASD had mothers with GDM 22.7% of controls had mothers with GDM OR: 2.30 (1.36–3.91)	Selection bias: Representativeness: 2 Participation rates: 2 Measurement bias: GDM: 2 ASD: 2	High
Kong et al. 2018^29^	Prospective cohort (population-based)	Finland	101,696 children of GDM mothers 543,347 children of non-diabetic mothers Aged up to 11 years	Medical records Diagnostic criteria unknown	Diagnosis from medical records	HR separated by BMI: Normal: 1.06 (0.88–1.28) Overweight: 1.27 (1.06- 1.52) Obese: 1.56 (1.26–1.93) Severely obese: 1.37 (1.04–1.81)	Selection bias: Representativeness: 0 Participation rates: 0 Measurement bias: GDM: 1 ADHD: 0	Low to moderate
Krakowiak et al. 2012^57^	Case control	USA	517 children with ASD 315 control children Aged 2–5 years	Medical records or self-report	Diagnosis by diagnostic interview	8.5% of children with ASD had mothers with GDM 6.0% of control children had mothers with GDM p = 0.18	Selection bias: Representativeness: 0 Participation rates: 2 Measurement bias: GDM: 2 ASD: 0	High
Li et al. 2016^58^	Prospective cohort	USA	102 children with ASD 1748 typically developing children Median age 67 months	Medical records Diagnostic criteria unknown	Diagnosis from medical records	8.8% of children with ASD had mothers with GDM 4.5% of typically developing children had mothers with GDM HR for ASD in GDM vs no diabetes: 1.86 (0.92–3.76) p = 0.08 HR for ASD in GDM and obesity vs neither condition: 3.04 (1.21–7.63) p = 0.02	Selection bias: Representativeness: 1 Participation rates: 2 Measurement bias: GDM: 1 ASD: 0	High
Maramara et al. 2014^30^	Retrospective cohort	USA	268 children with autism 115,632 control children from general New Jersey population Age of children not reported	Self-report, validated by medical records Diagnostic criteria unknown	Diagnosis reported from paediatric neurologist	4.7% of children with ASD had mothers with GDM 4.2% of the general New Jersey population had mothers with GDM P value not significant (not reported)	Selection bias: Representativeness: 0 Participation rates: 0 Measurement bias: GDM: 2 ASD: 0	High
Raz et al. 2015^59^	Case control	USA	245 children with ASD 1522 control children Age of children not reported	Self-report Diagnostic criteria unknown	Diagnosis from maternal report (validated in 50 cases)	7% of children with ASD had mothers with GDM (missing data for 16%) 6% of control children had mothers with GDM (missing data 14%)	Selection bias: Representativeness: 2 Participation rates: 0 Measurement bias: GDM: 2 ASD: 1	High
Sacks et al. 2016^35^	Prospective cohort (population-based)	Israel	12,642 children of GDM mothers 218,629 children of non-diabetic mothers Age of children not reported	Medical records Diagnostic criteria unknown	Diagnosis from medical records	0.04% children of GDM mothers had ASD 0.01% children of non-diabetic mothers had ASD Adjusted OR: 4.44 (1.55–12.69) p = 0.005 (Adjusted for maternal age, obesity, pre-eclampsia, fertility treatment, gestational week and time to event)	Selection bias: Representativeness: 1 Participation rates: 0 Measurement bias: GDM: 1 ASD: 0	Low to moderate
Say et al. 2016^60^	Retrospective cohort	Turkey	100 children with ASD Mean age 8.7 years (SD 3.86) 80 control children Mean age 8.5 years (SD 4.61)	Self-report Diagnostic criteria unknown	Diagnosis from expert child and adolescent psychiatrist	3% of ASD group were exposed to GDM 1.3% of control group were exposed to GDM p = 0.717	Selection bias: Representativeness: 2 Participation rates: 2 Measurement bias: GDM: 2 ASD: 0	High
Straughen et al. 2017^61^	Case control	USA	55 children with ASD 199 control children Age of children not reported	Medical records Diagnostic criteria unknown	Diagnosis from medical records	10.9% of children with ASD had mothers with GDM (7.2% unknown) 7.0% of control children had mothers with GDM (3.5% unknown)	Selection bias: Representativeness: 0 Participation rates: 0 Measurement bias: GDM: 1 ASD: 0	Low to moderate
Xiang et al. 2015^38^	Retrospective cohort	USA	25,035 children with GDM mothers 290,792 children with non-GDM mothers Age of children not reported	Medical records Diagnostic criteria: plasma glucose ≥ 200 mg/dL on glucose challenge test or defined by Carpenter-Coustan criteria on 100 g or 75 g OGTT	Diagnosis from medical records	1.2% of children of GDM mothers had autism 1.0% of children of non-GDM mothers had autism HR: 1.18 (1.04–1.33) p = 0.01	Selection bias: Representativeness: 0 Participation rates: 0 Measurement bias: GDM: 0 ASD: 0	Low to moderate

*%* percentage,* SD* standard deviation, *OR* odds ratio, *HR* hazard ratio, *RR* risk ratio, *β* β coefficient, *CI* confidence interval.

*Calculated by subtracting data for mothers with pregestational diabetes from data for non-GDM mothers.

### Pooled odds and prevalence of ASD in those exposed to GDM

Data on prevalence of ASD in those exposed to GDM were available for 15 studies; there were three studies which measured ASD and GDM but from which prevalence data could not be extracted^28–30^ (see Table 1). Heterogeneity on meta-analysis of these 15 studies was 98%, precluding meta-analysis. Median prevalence was 16.3% (IQR 0.9–48.8%).

**Figure 3:**
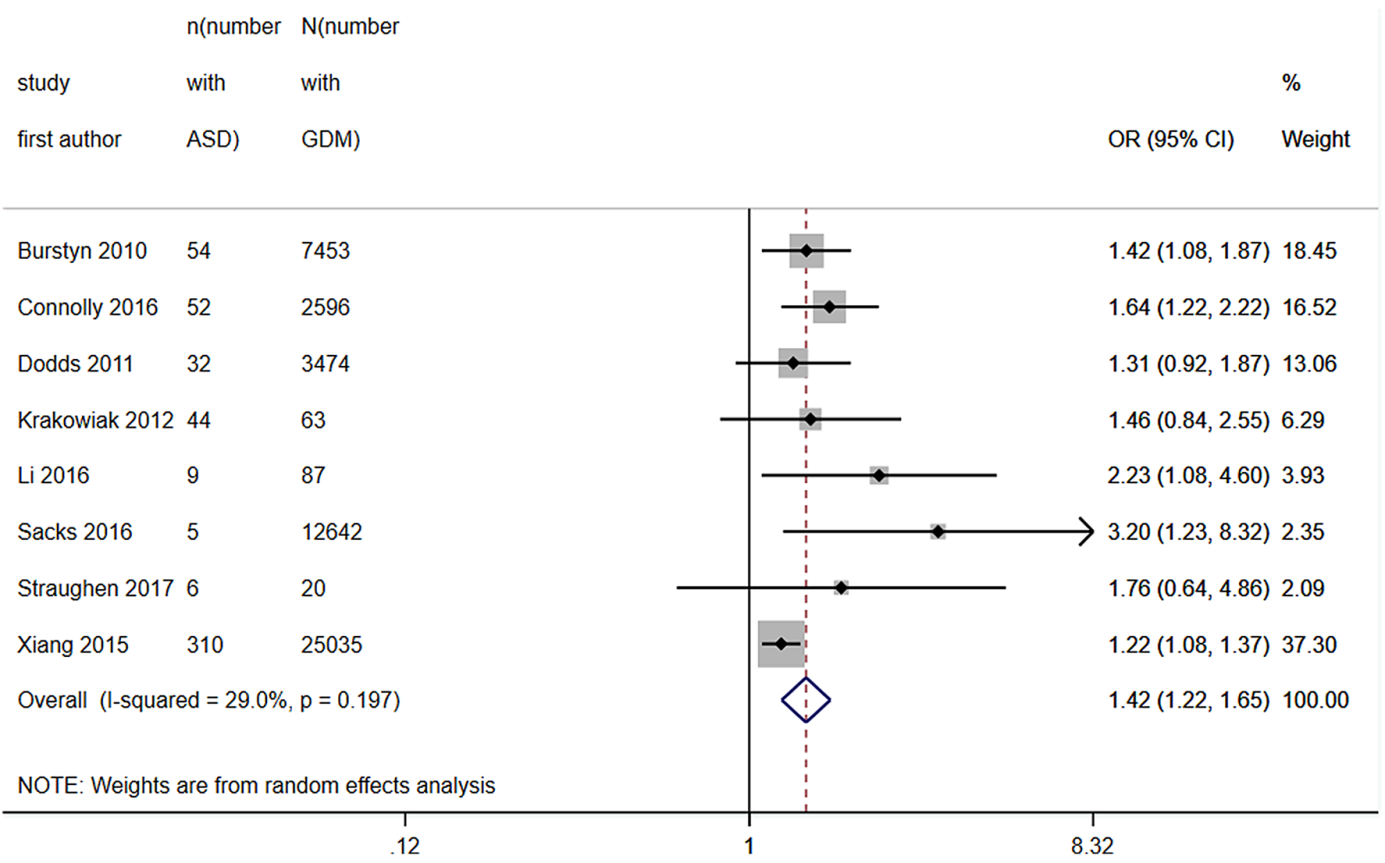
Forest plot showing pooled unadjusted odds ratios for ASD in those exposed to GDM versus those not exposed to GDM.

Eight of these studies were included in a meta-analysis of ORs. Studies were excluded in the absence of a control group or if there was doubt that pregestational diabetes had been excluded from the control group (not specifically mentioned in the exclusion criteria and no response to an e-mail to clarify). Pooled unadjusted OR was 1.42 (95% CI 1.22, 1.65) with heterogeneity 29% (see Fig. 3).

#### Characteristics of ADHD studies

15 studies measuring ADHD were identified. Table 2 provides a summary of their characteristics and findings. Compared to those studies measuring ASD, more of these studies were from European countries. All studies were observational; ten were prospective cohorts but there were less population-based cohorts than in the ASD literature so sample sizes were generally smaller. Moreover, measurement of symptoms using questionnaires were more frequently used in the ADHD literature (as opposed to diagnoses) and this in part led to only two studies being assessed as low to moderate risk of bias.

**Table 2:** Summary of studies measuring ADHD

Author and year	Study design	Country	Sample size and age of children at diagnosis	Ascertainment of GDM diagnosis	ADHD measure	Findings [%, mean (SD), OR/HR/RR, β (95% CI)]	Risk of bias score (low–high: 0–2)	Risk of bias
Akaltun et al. 2019^62^	Case control	Turkey	88 children of GDM mothers 128 children of non-diabetic mothers Aged 6–12 years	Medical records and self-report Diagnostic criteria defined by Carpenter-Coustan on 100 g OGTT	Diagnosis from medical records and parental report	GDM group: 15.9% had ADHD Non-GDM group: 7.0% had ADHD p = 0.115	Selection bias: Representativeness: 2 Participation rates: 2 Measurement bias: GDM: 0 ADHD: 0	High
Chiu et al. 2009^63^	Cross-sectional	Taiwan	11 children of GDM mothers 1380 children of non-GDM mothers Aged 4–9 years	Self-report Diagnostic criteria unknown	Symptoms of inattention using child behaviour checklist	OR not significant. No values given	Selection bias: Representativeness: 1 Participation rates: 0 Measurement bias: GDM: 2 ADHD: 2	High
Daraki et al. 2017^64^	Prospective cohort	Greece	55 children of GDM mothers 636 children of non-GDM mothers Aged 4 years	Medical records Diagnostic criteria defined by Carpenter-Coustan on OGTT	Symptoms using attention deficit hyperactivity disorder test (ADHDT)	β: 1.75 (-2.14,5.66) (Adjusted for child sex and age) β: 2.41 (95% CI: -1.45,6.28) (Further adjusted for maternal age, maternal origin, maternal education level, parity and maternal smoking during pregnancy) β: 2.32 ( -1.52,6.16) (Further adjusted for maternal pre-pregnancy BMI)	Selection bias: Representativeness: 0 Participation rates: 1 Measurement bias: GDM: 0 ADHD: 0	Low to moderate
Galera et al. 2018^65^	Prospective cohort	France	84 children of GDM mothers 1158 children of non-diabetic mothers Aged 3–8 years	Medical records or parental interviews Diagnostic criteria unknown	Symptoms by the Strengths and Difficulties Questionnaire	GDM group: 15.5% had high hyperactivity-impulsivity trajectories Non-GDM group: 14.9% had high hyperactivity-impulsivity trajectories	Selection bias: Representativeness: 0 Participation rates: 2 Measurement bias: GDM: 2 ADHD: 2	High
Kong et al. 2018^29^	Prospective cohort (population-based)	Finland	101,696 children of GDM mothers 543,347 children of non-diabetic mothers Aged up to 11 years	Medical records Diagnostic criteria unknown	Diagnosis from medical records	HR separated by BMI: Normal: 1.15 (1.01–1.30) Overweight: 1.16 (1.02- 1.32) Obese: 1.64 (1.42–1.88) Severely obese: 2.15 (1.84–2.52)	Selection bias: Representativeness: 0 Participation rates: 0 Measurement bias: GDM: 1 ADHD: 0	Low to moderate
Li et al. 2016^58^	Prospective cohort	USA	301 children with ADHD 1748 typically developing children Age of children not reported	Medical records Diagnostic criteria unknown	Diagnosis from medical records	4.0% of children with ADHD had mothers with GDM 4.5% of typically developing children had mothers with GDM HR for ADHD in GDM vs no diabetes: 0.99 (0.50–1.94) p = 0.98 HR for ADHD in GDM and obesity vs neither condition: 1.20 (0.49–2.93) p = 0.70	Selection bias: Representativeness: 1 Participation rates: 2 Measurement bias: GDM: 1 ASD: 0	High
Mina et al. 2017^43^	Prospective cohort	UK	14 children of GDM mothers 96 children of non-GDM mothers Aged 3–5 years	Medical records Diagnostic criteria unknown	Symptoms using Conners hyperactivity scale	Mean score for GDM children: 8.7 (6.0)* Mean score for non-GDM children: 7.3 (4.5)*	Selection bias: Representativeness: 2 Participation rates: 2 Measurement bias: GDM: 1 ADHD: 0	High
Nomura et al. 2012^44^	Prospective cohort	USA	21 children of GDM mothers Mean age 4.4 years (SD 0.48) 191 children of non-GDM mothers Mean age 4.3 years (SD 0.47)	Self-report Diagnostic criteria unknown	Diagnosis using a psychiatric interview and symptoms using ADHD rating scale-IV	Mean inattention score at baseline (no standard deviations given): GDM group: 12.25 Non-GDM group: 9.50 p = 0.05 Mean hyperactivity/ impulsivity scores: GDM group: 12.58 Non GDM group: 11.29 p = 0.36 76.2% children exposed to GDM had ADHD* 61.3% children not exposed to GDM had ADHD* OR at baseline: 1.58 (0.77–3.27) p = 0.22 OR at age 6 years: 2.20 (1.00–4.82) p = 0.05	Selection bias: Representativeness: 2 Participation rates: 2 Measurement bias: GDM: 2 ADHD: 0	High
Ornoy et al. 1999^66^	Prospective cohort	Israel	32 children of GDM mothers Mean age 8.5 years (SD 2.1) 57 children of non-diabetic mothers Mean age 8.3 years (SD 1.7)	Medical records Diagnostic criteria: abnormal glucose tolerance test (≥ 190 mg % glucose at 90 min, ≥ 165 mg % at 120 min, ≥ 145 mg % at 180 minutes, or with ≥ 105 mg % fasting glucose blood concentrations)	Symptoms using Conners parents’ questionnaire and Pollack Tapper Test	Conners parents’ questionnaire: Children of GDM mothers had mean score of 8.0 (6.5) if young and 6.8 (6.3) if older Control children had a score of 7.9 (4.3) if young and 7.0 (4.3) if older 4 GDM children had abnormal scores (above 14) compared with only 2 controls. p = 0.06 Pollack general: Children of GDM mothers had a mean score of 19.0 (12.4) if young and 29.6 (10.5) if older Control children had a score of 28.0 (3.2) if young and 30.3 (6.9) if older Pollack’s sound: Children of GDM mothers had a score of 10.6 (6.6) if young and 14.9 (5.0) if older Control children had a score of 14.8 (6.5) if young and 15.6 (3.6) if older Pollack visual Children of GDM mothers had a score of 7.7 (5.9) if young and 14.1 (5.4) if older Control children had a score of 13.2 (2.0) if young and 14.7 (3.4) if older	Selection bias: Representativeness: 2 Participation rates: 0 Measurement bias: GDM: 0 ADHD: 0	High
Pohlabeln et al. 2017^67^	Prospective cohort	Eight European countries	435 children of GDM mothers 18% under 4 years of age, 27% aged 4–6, 38% aged 6–8 and 17% over 8 years of age	Self-report Diagnostic criteria unknown	Diagnosis from parental report	1.8% of children exposed to GDM had ADHD 1.1% of children not exposed to GDM had ADHD OR: 1.42 (0.69–2.95) (adjusted for sociodemographics and country) OR: 1.28 (0.59–2.80) (further adjusted for pre-, peri- and postnatal influences) Unadjusted OR excluding pregestational diabetes: 1.149 (0.468–2.818) * Adjusted OR excluding pregestational diabetes: 1.032 (0.417–2.556) (adjusted for sex, age and country) *	Selection bias: Representativeness: 0 Participation rates: 0 Measurement bias: GDM: 2 ADHD: 1	High
Say et al. 2016^60^	Retrospective cohort	Turkey	100 children with ADHD Mean age 8.8 years (SD 1.98) 80 control children Mean age 8.5 years (SD 4.61)	Self-report Diagnostic criteria unknown	Diagnosis from expert child and adolescent psychiatrist	2% of ADHD group were exposed to GDM 1.3% of control group were exposed to GDM p = 0.717	Selection bias: Representativeness: 2 Participation rates: 2 Measurement bias: GDM: 2 ADHD: 0	High
Schmitt and Romanos 2012^45^	Cross-sectional (population-based)	Germany	280 children of GDM mothers 13,208 children of non-GDM mothers Aged 3–17 years	Self-report Diagnostic criteria unknown	Diagnosis from parental report	8.6% of children of GDM mothers had ADHD 5.1% of children of non-GDM mothers had ADHD Unadjusted OR: 1.93 (1.26–2.95) Adjusted OR: 1.91 (1.21–3.01) (adjusted for child sex, age, socioeconomic position, maternal smoking during pregnancy, maternal alcohol consumption during pregnancy, perinatal health problems, breastfeeding and atopic eczema)	Selection bias: Representativeness: 0 Participation rates: 1 Measurement bias: GDM: 2 ADHD: 1	High
Veena et al. 2010^32^	Prospective cohort	India	32 children of GDM mothers 515 children of non-diabetic mothers Aged 9–10 years	Medical records Diagnostic criteria defined by Carpenter-Coustan on OGTT	Symptoms using Coding-Wechsler Intelligence Scale for Children—3rd Edition (Coding WISC-III) score	Mean score for GDM group: 36.8 (8.0) Mean score for non-GDM group: 32.4 (8.1) p = 0.003 β: 0.4 (0.09–0.75) p = 0.01 (adjusted for child’s sex, gestation and age) β: 0.3 (0.01, 0.67) p = 0.04 (further adjusted for SES, parents’ education and rural/urban residence) β: 0.3 (-0.04, 0.67) p = 0.08 (further adjusted for maternal age, BMI and parity in pregnancy and child’s weight and head circumference at birth)	Selection bias: Representativeness: 0 Participation rates: 0 Measurement bias: GDM: 0 ADHD: 2	High
Wolford et al. 2017^31^	Prospective cohort	Finland	176 children of GDM mothers 1,603 children of non-GDM mothers (9 with T1DM) Mean 3.8 years (SD 0.5)	Medical records Diagnostic criteria unknown	Symptoms using Conners hyperactivity index (CHI)	Mean difference in CHI sum score for GDM vs no GDM: -0.05 (p = 0.08)	Selection bias: Representativeness: 0 Participation rates: 1 Measurement bias: GDM: 1 ADHD: 0	Low to moderate
Xiang et al. 2018^36^	Retrospective cohort	USA	29,534 children of GDM mothers 295,304 children of non-diabetic mothers Age of children not reported	Medical records Diagnostic criteria: ≥ 200 mg/dL blood glucose on 1 h 50 g glucose challenge test or 3 h 100 g or 2 h 75 g OGTT defined by Carpenter-Coustan criteria	Diagnosis from medical records	4.8% children of GDM mothers diagnosed with ADHD 5.2% children of non-diabetic mothers diagnosed with ADHD HR: 0.94 (0.88–1.00) p = 0.04 (adjusted for random sibling effect and birth year) HR: 1.02 (0.96–1.09) p = 1.50 (further adjusted for maternal age at delivery, parity, education, household income, maternal race/ethnicity, history of comorbidity, history of maternal ADHD and sex of the child) HR: 1.00 (0.94–1.06) (further adjusted for smoking, alcohol and pre-pregnancy BMI) HR: 1.01 (0.95, 1.08) (Adjusted for variables in first multivariate adjusted HR (1.02), plus pre-eclampsia, eclampsia, congenital anomalies, birth weight, and gestational age at delivery)	Selection bias: Representativeness: 1 Participation rates: 0 Measurement bias: GDM: 0 ADHD: 0	Low to moderate

*%*: percentage, *SD* standard deviation, *OR* odds ratio, *HR* hazard ratio, *RR* risk ratio, *β* β coefficient, *CI* confidence interval.

*Data provided by study author

#### Pooled odds and prevalence of ADHD in those exposed to GDM

Data on prevalence of ADHD in those exposed to GDM were available for eight studies. Heterogeneity on meta-analysis of prevalence from these eight studies was 93.7%, precluding meta-analysis. Median prevalence was 14.4% (IQR 6.7–41.3%).

Studies not included in meta-analysis were those presenting numerical scores on a symptom-based questionnaire, as opposed to numbers scoring above or below a defined threshold, precluding the calculation of prevalence or odds (see Table 2). Relatively small numbers of children are included in these studies, with only one study including over 100 children exposed to GDM^31^. All but one of these studies found no evidence of differences in scores between GDM exposed and unexposed children; one study found some evidence for greater concentration and inattention symptoms in children of mothers with versus those without GDM^32^, which was attenuated on adjustment for a range of obstetric, neonatal and sociodemographic confounders (see Table 2)..

**Figure 4:**
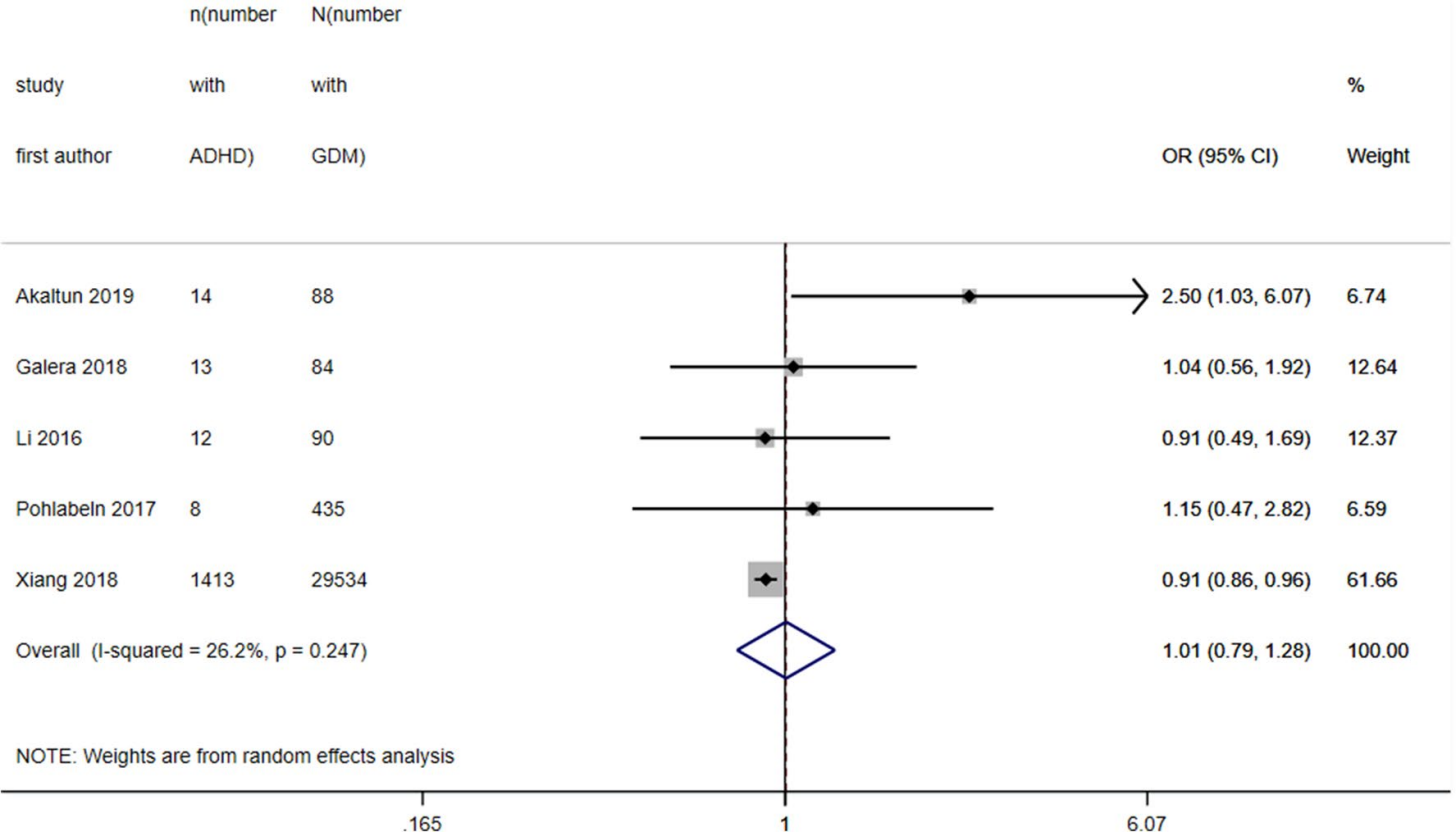
Forest plot showing pooled unadjusted odds ratios for ADHD in those exposed to GDM versus those not exposed to GDM.

Five of the eight studies providing information on prevalence were included in a meta-analysis of ORs; three studies were excluded for the same reasons as in the ASD meta-analysis i.e. unable to verify that pregestational diabetes had been excluded from the control population. Pooled unadjusted OR was 1.01 (95% CI 0.79, 1.28) with heterogeneity 26.2% (see Fig. 4).

#### Sensitivity analyses

In the meta-analysis of ORs for ASD, it appeared that effect sizes were slightly larger for those studies at low to moderate risk of bias. However, removal of the two studies at high risk of bias from the meta-analysis resulted in little change. Indeed, the pooled OR was slightly reduced at 1.39 (95% CI 1.19, 1.63). There were insufficient numbers of studies to facilitate the same sensitivity analysis for ADHD.

## Discussion

### Main findings

Pooled OR for risk of ASD following exposure to GDM was 1.42 (95% CI 1.22, 1.65) and for ADHD was 1.01 (0.79, 1.28). In general, studies measuring ADHD more often utilised screening tools of symptoms in smaller populations than the studies measuring ASD, which more often measured clinical diagnoses within larger population-based cohorts. Median prevalence of ASD of 16.3% and ADHD of 14.4% in those exposed to GDM is higher than that estimated in the general population^15,33^. However, there was substantial heterogeneity between studies included in these estimates, also reflected in wide IQRs for these medians and indicative of the broad range of study designs, populations and measures of both exposure and outcomes. That ORs in these studies when comparing risk in the GDM exposed versus unexposed were only modestly elevated also suggests that rates of ASD and/or ADHD were elevated in the study population as a whole, either due to selection of at-risk samples or due to systematic measurement of symptoms. Nonetheless, pooled unadjusted OR for risk of ASD in those exposed to GDM of 1.42 provides some evidence for a slightly increased risk, not seen to the same extent for ADHD (OR 1.01). Two previous meta-analyses investigating only risk for ASD found an increased risk; one with a pooled relative risk (RR) of 1.63 had substantially more heterogeneity (I^2^ 75%)^7^ and the other with RR between 1.48 and 1.72 did not separate pregestational and gestational diabetes^6^. In contrast to our meta-analysis, in a meta-analysis of risk for ADHD following exposure to GDM across four studies, RR was 2.0 (95% CI 1.42, 2.81)^5^. However, as previously discussed, these meta-analyses did not specifically exclude pregestational diabetes from their control populations, which may explain the difference in results.

### Strengths and limitations

This is the first study to our knowledge that has rigorously reviewed the literature and meta-analysed prevalence and risk from studies pertaining to both diagnoses and symptoms of ASD and ADHD. Using the same review strategy for more than one neurodevelopmental disorder allows a direct comparison of risk across a range of disorders. Another unique strength of this review is the exclusion from meta-analysis those studies in which pregestational diabetes was not removed from the control population. As previously discussed, pregestational and gestational diabetes differ somewhat in their pathology which could have implications for the degree of risk for adverse neurobehavioural outcomes and potential mechanistic pathways discussed below. However, just as the degree of glucose intolerance may differ between pregestational and gestational diabetes, it can also differ between populations with GDM due to widespread variation in diagnostic criteria. A significant limitation of the studies included within this review is that most of them do not provide information on GDM diagnostic criteria or any other indicators of GDM severity such as use of insulin or medication. Yet there is now evidence that maternal hyperglycaemia even below that of diagnostic threshold for GDM may be associated with adverse obstetric and neonatal outcomes^34^. It may be useful for future studies to investigate the impact of severity of maternal hyperglycaemia on risk for neurodevelopmental disorders; for example, whether or not there is a dose response relationship between maternal glucose levels and risk for disorder.

Lack of reporting on GDM diagnostic criteria within the included studies is one of the reasons why over half of the studies were assessed as at high risk of bias, although removal of studies at high risk of bias in the ASD meta-analysis of ORs resulted in minimal change to the effect estimate. However, there was also substantial diagnostic heterogeneity in the outcome of neurodevelopmental disorders, particularly in ADHD, where a broad range of questionnaires measuring levels of symptoms of ADHD were measured, which may not have met diagnostic threshold. Insufficient numbers of studies were available for ADHD to explore the impact that this may have had within a sensitivity analysis. A further limitation of the studies included within this review is that only some investigated the influence of other factors on the GDM and neurodevelopmental disorders association. This is discussed further below.

### Potential mechanisms

The differences in risk between ASD and ADHD found in this review could be due to differing causal pathways, although clearly there are limitations to inferring any causality from observational studies. It could also be due to differences in the exposure, specifically degree of hyperglycaemia, although as previously discussed, this is often difficult to assess as so few studies consider it. Another possibility is that smaller sample sizes in the ADHD studies have failed to provide sufficient power to detect a difference in risk.

There were a few studies which looked at possible indicators of severity of GDM and degree of hyperglycaemia. For example, studies comparing GDM treated with medication versus without suggested an increased risk in medication-treated groups for both ASD^35^ and ADHD^36^. The pathway through which hyperglycaemia may impact neurodevelopment may be mediated by oxidative stress, which has been associated with adverse neurobehavioural outcomes such as motor deficits^13^. It may also influence epigenetic changes in the offspring, such as reduced DNA methylation seen in neurodevelopmental disorders such as ASD^14^. Moreover, hyperglycaemia can lead to systemic inflammation and pro-inflammatory cytokines are able to cross the placenta and the foetal blood–brain barrier, which may affect neurodevelopment^37^. However, there may be critical periods of exposure to hyperglycaemia during pregnancy for the different neurodevelopmental conditions. Xiang et al. have conducted analyses in a large population-based cohort on risk for both ASD^38^ and ADHD^36^ following exposure to maternal diabetes. They found that the later the GDM is diagnosed, the lesser the risk of ASD but saw no association with ADHD which may indicate differing critical periods.

Women with GDM are at a greater risk of several adverse obstetric outcomes, such as pre-eclampsia, foetal macrosomia, perinatal mortality, Caesarean delivery and preterm delivery^9,39^, which may also increase the risk of neurodevelopmental disorders^40^. While some studies presented data on gestational age at birth and birthweight, none explored their role as a potential mediator. A number of studies also investigated the role of obesity and socioeconomic status (SES) as effect modifiers of the association between GDM and neurodevelopmental disorders. Higher body mass index (BMI) increased the risk of both ASD^29,41^ and ADHD^29,42,43^ following exposure to GDM. Likewise, low SES has been shown to further increase the risk of ADHD^44,45^ following exposure to GDM, although this has been less explored in ASD.

### Implications and conclusions

Therefore, future potential areas for research include an investigation of these mechanistic pathways underlying the association between maternal hyperglycaemia across the spectrum of subclinical, gestational and pregestational diabetes, and adverse neurobehavioural outcomes. Baseline risk for neurodevelopmental disorders in the general population is relatively low so absolute risk for a neurodevelopmental disorder in the offspring of mothers with GDM is still relatively low and there are many children exposed to GDM during pregnancy who do not develop a neurodevelopmental disorder. This supports an approach to measuring risk on a continuum and is one of the reasons that we chose to include symptoms of disorder in addition to clinical diagnoses.

A greater understanding of the early determinants of a child’s cognitive, social and emotional wellbeing would add support to interventions aimed at better management of these adversities, such as GDM, during pregnancy. Access to information about their condition has been identified as an enabler for women with GDM to manage their condition^46^. Such information could include sensitively informing women about potential risks to their baby. There is now evidence that effective management results in reductions in obstetric morbidities such as shoulder dystocia and pre-eclampsia^47^. However, there is also some evidence to support an inverse correlation between level of hyperglycaemia in pregnancy and longer-term neurobehavioral outcomes in offspring, such as verbal IQ^48^.

Furthering knowledge of these early predictors of adverse neurobehavioural outcomes would also underscore the importance of interventions aimed at prevention of such adverse pregnancy exposures by targeting their broader determinants in early pregnancy or even earlier in the preconception period. For example, there is some evidence that diet and physical activity interventions in early pregnancy reduce gestational weight gain and may be associated with a reduced risk of GDM^49^. That a number of studies included in the review found that socioeconomic status was an effect modifier of the association between GDM and neurodevelopmental disorders also highlights the importance of considering the broader determinants of health within healthcare. Thus there are a number of points at which healthcare professionals and policy makers involved in the care of women and children affected by GDM may usefully intervene.

In conclusion, there may be an association between GDM and the neurodevelopmental disorders of ASD and ADHD, with potentially differing levels of risk and mechanistic pathways for different neurodevelopmental disorders. Greater understanding of these risks and mechanisms may help to modify potential adverse developmental trajectories from becoming established in children.

## Supplementary Information

Supplementary Information.
