# Supplementary material for: The association between gestational diabetes and ASD and ADHD: a systematic review and meta-analysis
Source: Sci Rep. 2021 Mar 4;11:5136. doi: 10.1038/s41598-021-84573-3 (PMC7933135; doi:10.1038/s41598-021-84573-3)
Supplement: Supplementary file 1 — Supplementary Information. [file 41598_2021_84573_MOESM1_ESM.pdf]

Search terms for ASD

1. MeSH terms: diabetes, gestational (in PubMed and The Cochrane Library), diabetes mellitus, gestational (in CINAHL), gestational diabetes (in PsycINFO), pregnancy diabetes mellitus (in EMBASE).
2. "Gestational diabetes"
3. Gestation* adj2 diabet*
4. Glucose adj4 (pregnan* or gestation*)
5. Insulin near (pregnan* or gestation*)
6. Insulin adj4 (pregnan* or gestation*)
7. GDM
8. 1 OR 2 OR 3 OR 4 OR 5 OR 6 OR 7
9. MeSH terms: autistic disorder OR autism spectrum disorder OR asperger syndrome (in PubMed and The Cochrane Library), autistic disorder OR asperger syndrome (in CINAHL), autism spectrum disorders (in PsycINFO), autism OR asperger ayndrome (EMBASE).
10. Autis*
11. Asperg*
12. ASD
13. 9 OR 10 OR 11 OR 12
14. 8 AND 13

Search terms for ADHD

1. MeSH terms: diabetes, gestational (in PubMed and The Cochrane Library), diabetes mellitus, gestational (in CINAHL), gestational diabetes (in PsycINFO), pregnancy diabetes mellitus (in EMBASE).
2. “Gestational diabetes”
3. Gestation* adj2 diabet*
4. Glucose adj4 (pregnan* or gestation*)
5. Insulin near (pregnan* or gestation*)
6. Insulin adj4 (pregnan* or gestation*)
7. GDM
8. 1 OR 2 OR 3 OR 4 OR 5 OR 6 OR 7
9. MeSH terms: attention deficit disorder with hyperactivity (in PubMed and The Cochrane Library), attention deficit hyperactivity disorder (in CINAHL), attention deficit disorder (in EMBASE), attention deficit disorder with hyperactivity OR attention deficit disorder (in PsycINFO).
10. “Attention deficit hyperactivity disorder”
11. Attention-deficit
12. Attention deficit
13. Inattent*
14. Hyperactiv*
15. “Hyperkinetic disorder”
16. Hyperkine*
17. Hyper near activ*
18. Attention near deficit
19. ADHD
20. ADDH
21. HKD
22. 9 OR 10 OR 11 OR 12 OR 13 OR 14 OR 15 OR 16 OR 17 OR 18 OR 19 OR 20 OR 21
23. 8 AND 22

Risk of bias assessment tool

Question	Score (0-2: low to high risk of bias)	Comments
**Study type**		
What is the study type?<br>0- Prospective cohort, interventional or population-level<br>1- Case control, retrospective cohort or cross-sectional<br>2- Unknown		
**Selection bias**		
Representativeness of exposed cohort<br>0- Representative of the general population<br>1- Moderately limited in its representation of the general population<br>2- Only representative of a very specific population		
Participation rates (for cross-sectional studies)<br>0- 80-100% agreed to participate or retrospective population-based data<br>1- 60-79% agreed to participate<br>2- Less than 60% agreed to participate or participation rates not reported<br>Follow-up/drop out in study (for longitudinal studies)<br>0- Drop-out rate of <40%<br>1- Drop-out rate of 40-59%<br>2- Drop-out rate of 60% or higher/not reported		
**Measurement bias**		
Ascertainment of GDM exposure<br>0- Diagnosis reported from medical professional/medical notes with diagnostic criteria<br>1- Diagnosis reported from medical professional/medical notes without diagnostic criteria<br>2- Method of report unknown/self-report		
Assessment of outcome<br>0- ASD/ADHD diagnosis from medical professional or medical notes or symptoms from an ASD/ADHD questionnaire<br>1- Parental report of ASD/ADHD diagnosis<br>2- Method of report unknown or symptoms on a questionnaire not specifically focussed on ASD/ADHD		
**Comparability**		
Controlling for confounders<br>0- Maternal obesity and one or more other confounders controlled or adjusted for<br>1- Only maternal obesity controlled or adjusted for as a confounder, or several other factors excluding maternal obesity controlled for<br>2- No attempt to correct, control or adjust for confounding factors		
